# Complementary biobank of rodent tissue samples to study the effect of World Trade Center exposure on cancer development

**DOI:** 10.1186/s12967-019-2089-7

**Published:** 2019-10-11

**Authors:** Wil Lieberman-Cribbin, Stephanie Tuminello, Christina Gillezeau, Maaike van Gerwen, Rachel Brody, David J. Mulholland, Lori Horton, Maureen Sisco, Colette Prophete, Judith Zelikoff, Hyun-Wook Lee, Sung-Hyun Park, Lung-Chi Chen, Mitchell D. Cohen, Emanuela Taioli

**Affiliations:** 1 Institute for Translational Epidemiology and Department of Population Health Science and Policy, Icahn School of Medicine at Mount Sinai, One Gustave L. Levy Place, Box 1133, New York, NY 10029 USA; 2 Department of Pathology, Icahn School of Medicine at Mount Sinai, New York, NY USA; 3 Department of Medicine, Hematology and Medical Oncology, Icahn School of Medicine at Mount Sinai, New York, NY USA; 4 Nelson Institute of Environmental Medicine, New York University, Tuxedo Park, NY USA

**Keywords:** Rodents, World Trade Center Dust, Biorepository

## Abstract

World Trade Center (WTC) responders were exposed to mixture of dust, smoke, chemicals and carcinogens. New York University (NYU) and Mount Sinai have recreated WTC exposure in rodents to observe the resulting systemic and local biological responses. These experiments aid in the interpretation of epidemiological observations and are useful for understanding the carcinogenesis process in the exposed human WTC cohort. Here we describe the implementation of a tissue bank system for the rodents experimentally exposed to WTC dust. NYU samples were experimentally exposed to WTC dust via intratracheal inhalation that mimicked conditions in the immediate aftermath of the disaster. Tissue from Mount Sinai was derived from genetically modified mice exposed to WTC dust via nasal instillation. All processed tissues include annotations of the experimental design, WTC dust concentration/dose, exposure route and duration, genetic background of the rodent, and method of tissue isolation/storage. A biobank of tissue from rodents exposed to WTC dust has been compiled representing an important resource for the scientific community. The biobank remains available as a scientific resource for future research through established mechanisms for samples request and utilization. Studies using the WTC tissue bank would benefit from confirming their findings in corresponding tissues from organs of animals experimentally exposed to WTC dust. Studies on rodent tissues will advance the understanding of the biology of the tumors developed by WTC responders and ultimately impact the modalities of treatment, and the probability of success and survival of WTC cancer patients.

## Introduction

The World Trade Center (WTC) disaster resulted in a large cloud of dust containing a mixture of toxic chemicals such as soot, benzene/other volatile organic compounds, asbestos, silica, cement dust, glass fibers, heavy metals, polycyclic aromatic hydrocarbons (PAHs), polychlorinated biphenyls (PCBs), and polychlorinated dibenzofurans and dioxins [1–3].

The health consequences of exposure to these chemicals were both acute and chronic [4–6]. In the immediate aftermath and for months/years thereafter, first responders and others suffered from a variety of health issues, including upper airway inflammation disorders, asthma, and cough [7]. Long-term follow-up of WTC responders has shown an increase in cancer incidence over time [8–10], possibly related to the presence of multiple known/suspected human carcinogens in the dust cloud [11–17]. This observation prompted the creation of a solid cancer tissue bank to supplement the current epidemiologic studies on cancer occurrence [18]. Establishing a central repository of these tissues allows investigators to better elucidate connections between WTC dust exposures and cancer etiology/progression among first responders in comparison to other individuals with cancer who were not exposed to the WTC dusts.

The laboratories of Drs. Cohen and Chen at the Department of Environmental Medicine at the New York University (NYU) School of Medicine are the only one known to have Ground Zero dusts collected within 72 h after the towers collapsed. These samples are unique in that all other WTC dust samples known to have been collected were gathered after a significant rainstorm hit New York City on 9/14/01 or thereafter. No collected samples are known to exist of WTC dusts generated on 9/11/01 itself. Chemical analyses of the dusts’ contents yielded a wide variety of metals and concentration profiles within size classes, as well as organics such as PAHs and PCBs, with various forms of each class present at widely-varying levels: specific forms often only found at ≪ 0.1% of total bulk mass [11, 15, 19, 20]. Later studies of the dust found metal composition profiles consistent with the initial findings [21]. These studies combined with the findings of Lippman et al. [6] suggests that these dusts best represent the materials present in the air at Ground Zero that first responders were exposed to in the earliest time period after the disaster.

We are now implementing a tissue bank of rodents experimentally-exposed to unique samples of WTC dusts collected on-site in the first 2 days (i.e., 9/12/01–9/13/01) following the disaster. In addition, the biobank includes tissue from genetically modified mice exposed to WTC dust through nasal instillation at Mount Sinai for the purpose of studying the effect of WTC dust exposure on tumor progression.

## Methods

### Standardization of samples collection, preparation and storage

Since the biobank houses samples derived from experiments conducted in different laboratories, it was important that a common standard operating procedure be developed to assure that the samples were prepared and stored under a common protocol. The laboratories have exchanged protocols, worked on the standardization process, and shipped material to be centrally stored in the biobank. All processed tissues include annotations related to the experimental design, the WTC dust concentration/dose, the exposure route and duration, the genetic background of the rodent, and the method of tissue isolation/storage.

### New York University experiments

#### Cardiovascular and pulmonary endpoints in rodents

Several funded experiments at NYU have provided samples stored in the biobank. This includes samples from an ongoing study using spontaneously hypertensive rats (SHR) to evaluate mechanisms of dust exposure-associated hypertension-induced cardiac hypertrophy as it progresses to heart failure, damage to arteries, athero-/arteriosclerosis, and inducible changes in cardiovascular (CV) structure, function, and gene expression. This study seeks to discern how entrained particles, alone or in conjunction with another air pollutants present at Ground Zero such as diesel exhaust, might induce changes in the CV system of first responders. Potential novel biomarkers for CV diseases resulting from the complex exposure scenarios are also being studied for potential use in screening of “still healthy” WTC responders to identify any early onset of exposure-related CV diseases. The biobank also houses samples from a different strain of rats (F344) used to examine the effects of WTC dusts on a variety of lung-related parameters including changes in airway morphology, cell composition, and increased expression of genes related to lung inflammation [22, 23]. These studies were performed because pulmonary pathologies were a major health effect observed in the earliest time points among first responders.

#### New York University methodology

Rats in both NYU studies were exposed to coarse [10–53 µm] WTC dust that comprised > 90% of all the WTC dust present in the air in the immediate aftermath of the disaster. Control rats were exposed in parallel to either the anesthesia isofluorane (ISO, in oxygen) only, or air only (naïve control). Anesthesia exposure was necessary as exposure was administered via intratracheal inhalation, using a completely novel system build at NYU [21] that allowed for large particles like the WTC dusts to enter the lungs in a manner mimicking inhalation routes of first responders at Ground Zero. The use of this method also ensured WTC dusts were unadulterated, as might otherwise occur if exposure particles were suspended in a liquid vehicle. In both studies, rats were exposed for 2 h/day on 2 consecutive days to model the “reference responder” [5] and atmospheres likely faced by responders (i.e., ≈ 250 mg WTC dust/m^3^ during 11–13 September 2001) [24]. Rats were then euthanized at 1, 30, 60, 120, 240, and 360 days post-exposure and tissues were either immediately examined or harvested/stored and then shipped to Mount Sinai for storage in the biobank.

#### New York University mice experiments

The biobank also stores samples from WTC dust-exposed mice from experiments performed at NYU. Specific pathogen-free 8–10 week old male mice strains C57BL/6 and FVB/NJ were anesthetized (1–3% ISO in oxygen) and intranasally-exposed to suspended particles and their soluble components in single or multiple exposure events. Mice were euthanized 24 h after their final exposure. Serum, bronchoalveolar lavage fluid (BALF), lungs, liver, brain, and heart were collected and flash frozen in liquid N_2_, and stored at − 80 °C for later analysis. Mice exposed to WTC dust intranasally were injected intraperitoneally with dexamethasone (0.1 mg/kg) or Drug X (5 mg/kg; proprietary) immediately post-exposure. Lungs were then removed 24 h post-exposure, flash frozen in liquid oxygen, and stored at − 80 °C for later analysis. All animal procedures were conducted under protocols approved by New York University Institutional Animal Care and Use Committee (IACUC).

### Mount Sinai experiments

#### PTEN loss in prostate and lung tissue of mice

Several tissues types from mice exposed to WTC dust via nasal instillation at Mount Sinai are stored in the biobank. These mice are altered to have homozygous loss of the phosphatase and tensin homologue (PTEN) gene [25, 26], which acts as a tumor suppressor and is important for cell proliferation, among other functions [27, 28]. Both heterozygous and homozygous PTEN loss has been observed in a variety of cancers and autoimmune diseases [29–31], including prostate [28, 32–37] and lung cancer [38, 39]. In mouse models, deletion of PTEN on both alleles promotes metastatic prostate cancer [40], further underscoring the role of PTEN in cancer development and progression. At Mount Sinai, mice with homozygous loss of PTEN specifically in the prostate and in the lung have been studied to better understand cancer development in the WTC exposed cohort. All procedures were conducted under a protocol approved by the Icahn School of Medicine IACUC.

#### Mount Sinai methodology

In these experiments, mice were treated with 3 doses (4 mg) of dust every other day via nasal instillation. In mice with biallelic deletion of PTEN in the prostate, all organs were harvested from 1 to 6 months after exposure, while organs from mice with PTEN (−/−) expression in lung tissue were harvested starting after 4 months. Prostate tissue was excised by grasping the bladder and cutting off the urethra. Seminal vesicles, the ampullary gland, the bladder, ureters, and urethra were all removed while the harvested organ was maintained in sterile phosphate-buffered saline (PBS, pH 7.4). The remaining prostate lobes (dorsal, ventral, lateral, anterior) were pooled and snap frozen in TRizol. For prostate fixation, resected tissues were (and will be) submersion-fixed in formalin (10% neutral buffered formalin, 1 mm/h) for up to 24 h.

Total lung tissue was resected, briefly dissected in PBS, and snap frozen in TRizol. For fixation of the lung, formalin was passed though the trachea using a syringe resulting in inflation and proper histological preparation [41]. Total spleen was/will be collected, washed gently in PBS, and snap frozen in TRizol.

Sera and peripheral blood mononuclear cells (PBMC) were separated from blood collected using a lancet (for mandibular bleeding) to quickly draw 0.4–0.5 ml of blood. The collected material was divided into two tubes, one for sera and one for PBMC isolation. To isolate sera, whole blood was allowed to clot for 30 min and then centrifuged for 15 min at 2500 revolutions per minute. To isolate PBMC, whole blood was treated with Ammonium–Chloride–Potassium red blood cell lysis buffer for 1 min, the cell suspension was then repeatedly washed with PBS, and the final pellet was then snap-frozen in TRizol. All isolated RNA samples were stored in a dedicated − 80 °C freezer. These tissue isolation approaches have been be used both for normal and cancer-induced WTC mouse models.

## Results

The central repository of bio-samples from rodents exposed to WTC dust exists for various organs and blood products. From NYU, this includes biosamples from male SHR rats exposed to ISO, air, or the indicated WTC dust concentrations collected at various intervals over a 1-year post-exposure period (Table 1). Organs from WTC dust-exposed mice are also centrally stored (Table 2). From Mount Sinai, this includes organs from mice with homozygous loss of PTEN expression in the prostate and in the lung (Table 3).

**Table 1:** Inventory of NYU bio-samples from WTC dust-exposed/control SHR rats

Harvest time post-exposure	Exposure type	Number of rats with organs and blood stored^a^
Day 1	Naïve	6
	ISO control	5
	26.5–35.5^b^ mg dust/m^3^	6
Day 30	Naïve	6
	ISO control	6
	26.6–32.0 mg dust/m^3^	6
Day 60	Naïve	6
	ISO control	6
	32.7–35.5 mg dust/m^3^	6
Day 120	Naïve	6
	ISO control	6
	32.7–35.5 mg dust/m^3^	6
Day 240	Naïve	12
	ISO control	6
	32.0–35.5 mg dust/m^3^	5
Day 360	Naïve	6
	ISO control	6
	26.6–42.8 mg dust/m^3^	17
Total		123

Tissues from the male F344 rats of the earlier intratracheal inhalation WTC dust-exposure studies (also 2 h/day on 2 consecutive days) are also available in the Biobank, although the group sizes are not presented here

^a^ All rats were males. Organs isolated for each rat included the heart, aortic arches, prostate, kidney, liver, thyroid, spleen; these materials were frozen and/or stored in formalin. TA leg muscle, plasma, serum, bone marrow and lung lavage fluid (supernatant) were only frozen. Some lungs were lavaged and then frozen; others were inflated and then fixed

^b^ Values shown cover range of dust levels presented to the rats over the course of their respective two exposures. Target value each day was ≈ 33–35 mg/m^3^. To avoid bias, rats from any given exposure set were randomly allocated into the various post-exposure time groups; this explains commonality among ranges indicated

**Table 2:** Inventory of NYU bio-samples from WTC dust-exposed mice

Route of exposure	Harvest date post-exposure (days)	Treatment	Storage format
IN	1, 7, 30, 90	None	Frozen
IN	1	Dexamethasone	Frozen
IN	1	Drug X	Frozen

Strain BL6 and FVBN mice were intranasally-instilled once with WTC dust. In all cases, bio-samples (i.e., heart, kidney, and liver) were harvested 1, 7, 30, and 90 days after exposure, snap-frozen, and stored at − 80 °C. In a parallel study, mice were IP-injected immediately after the exposure with either dexamethasone (0.1 mg/kg) or Drug X (proprietary; 5 mg/kg). I in this study, bio-samples were collected only 24 h post-exposure, and then stored

**Table 3:** Description of Mount Sinai bio-samples from WTC dust-exposed BL6 mice

Bio-sample	Genotype	Storage format
Genetic modification in prostate		
Prostate^a^	*Pten* ^− */*−^	Frozen + paraffin
Lung	*Pten* ^+ */*+^	Frozen + paraffin
Bladder	*Pten* ^+ */*+^	Frozen
Thyroid	*Pten* ^+ */*+^	Frozen
Spleen	*Pten* ^+ */*+^	Frozen
Whole blood	*Pten* ^+ */*+^	Frozen
Serum	*Pten* ^+ */*+^	Frozen
Genetic modification in lung		
Prostate	*Pten* ^+ */*+^	Frozen + paraffin
Lung^b^	*Pten* ^− */*−^	Frozen + paraffin
Bladder	*Pten* ^+ */*+^	Frozen
Thyroid	*Pten* ^+ */*+^	Frozen
Spleen	*Pten* ^+ */*+^	Frozen
Whole blood	*Pten* ^+ */*+^	Frozen
Serum	*Pten* ^− */*−^	Frozen

All mice were intranasally-instilled, 3 doses (4 mg) every other day. Time to harvest was 1–6 months post-final exposure in mice with genetically-modified prostates, and > 4 months post-final exposure in mice with genetically-modified lung tissue

^a^ *Pb*-*Cre*+; *PtenLL GEM model*

^b^ Mice treated with (1) WTC dust, (2) PBS, (3) WTC dust + Ad-cre, or (4) PBS + Ad-cre

## Discussion

We present here the first tissue bank of rodents derived from WTC exposure laboratory experiments. This rodent tissue bank will complement the existing tissue bank of human cancer samples from WTC responders [18]. Since 9/11, a main priority of the WTCHP has been to expand WTC surveillance programs, conduct follow-up, and report cancer incidence rates through linkage with cancer registries. Given the projected increased cancer burden among WTC-exposed Fire Department of the City of New York rescue and recovery workers, notably for prostate, thyroid, and melanoma [10], there is a need for a biorepository of cancer tissue to study cancer etiology and development and to assess if these cancers are biologically different from corresponding cancers that developed in subjects not exposed to WTC dusts.

However, as etiologic studies require the availability of properly prepared and stored tissues, the human tissue biobank of WTC samples is a necessity for existing and incident cancer cases. Although the number of cancer cases in the WTCHP is expected to increase given the latency period between exposure and cancer progression and the aging of the population, the amount of human tissue is still limited, and as such the tissue will be prioritized by the Utilization Committee in favor of research studies that show the most promising venue. However, the rodent tissue bank offers a more renewable resource for conducting studies that can then be translated to human cancer, with the ability to recreate exposure conditions to those following 9/11. Thus this biobank provides the opportunity to compare systemic and local changes observed in WTC dust-exposed rodents to what is observed in human cancer tissues and the peripheral blood of responders who developed cancer, as well as to normal tissue of those WTC responders who to date appear to still be healthy.

### Potential for translational research

Studying a variety of tissues and endpoints in these rodents increases the overall understanding of both the acute and chronic biological effects of exposure to WTC dusts, in addition to clarifying if and how WTC dusts impacted cancer initiation and/or progression in first responders. The results of these animal experiments have high translational potential, as any local and systemic biological response observed in rodents experimentally exposed to the WTC dusts could help in the interpretation of the epidemiological observations [42]. Further, findings in the rodents could drive the choices of the most appropriate biomarkers to assess the effects from the dust exposures in the WTC cohort.

As rodents in the biobank are exposed to different doses of WTC dust and for different time spans, available tissues will reflect and mimic both the intensity and the duration of exposure that occurred in WTC responders. This is especially valuable for studying health outcomes given the spectrum of WTC exposure experienced by first responders. While an exposure index for WTC responders exists, it was reconstructed from questionnaires where recall bias could affect accuracy of reporting during this time of extreme stress, further necessitating creating a spectrum of WTC exposure in rats to mimic human exposure conditions.

**Fig. 1:**
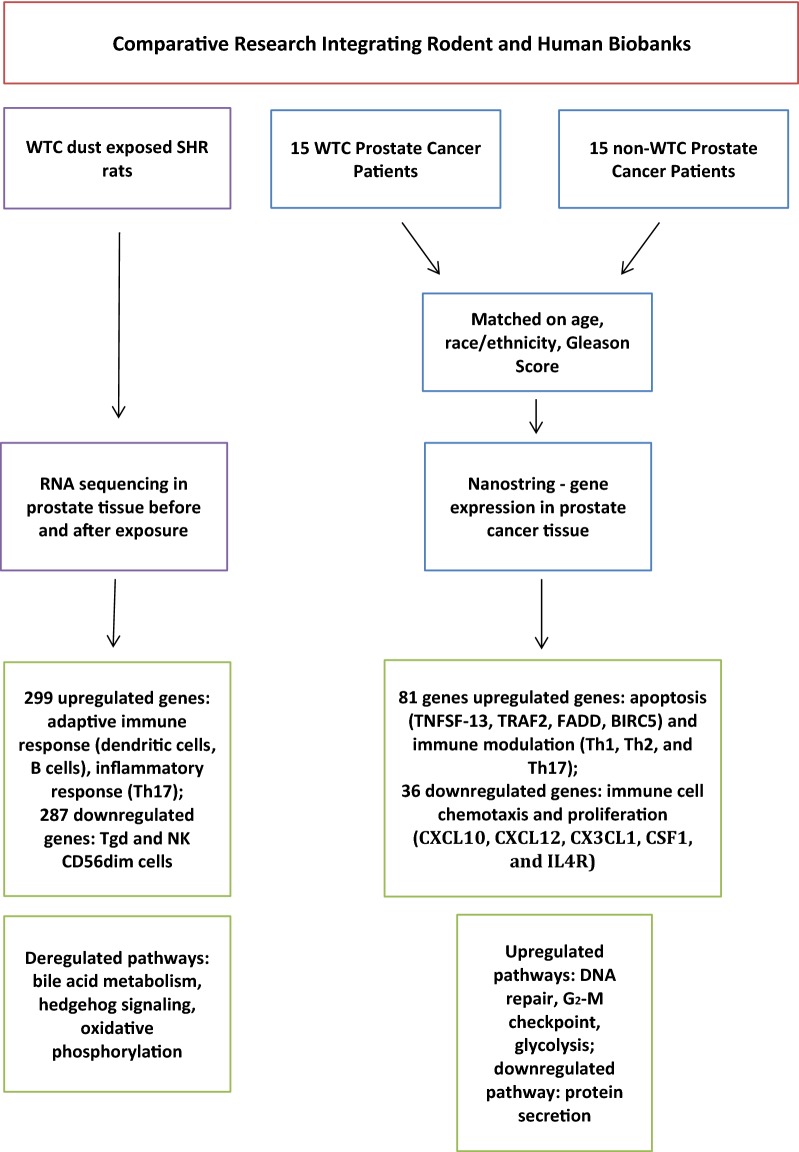
Comparative research using the human and animal biobank—the example of prostate cancer [42]

Several exposure studies in rodents have been planned and conducted to study the generalized and local immune and inflammatory response triggered by the WTC dusts. These same rats were also going to be evaluated to ascertain if the dust exposures stimulated tumor formation/progression. Specifically, given the increased risk of prostate cancer reported in WTC responders and the recovery workers cohort, a proof of principle study on the role of inflammation on prostate cancer using DNA and RNA sequencing was conducted using human material stored in the biobank. The study showed that WTC prostate cancer cases had upregulation of genes involved in DNA damage, cell division, and inflammatory response [42]. These results were then compared with the results observed in prostate tissue of rats exposed to WTC dust and stored in the biobank, reporting that WTC dust induces an inflammatory and adaptive immune response in prostate tissue of rats as well (Fig. 1).

As mentioned previously [18] genomic research, gene-environment interaction, and DNA methylation studies are needed to better understand the link between WTC exposure and clinical cancer characteristics [43, 44]. The WTCHP manages datasets containing comprehensive clinical information and details on environmental exposures that occurred at the WTC site. These datasets can be conveniently linked with the biobank of human and animal tissue, providing future investigators opportunities to study cancer genetic markers of aggressiveness, etiology, biology and outcomes in the WTC population. This will facilitate studies of the systemic inflammatory and immuno-response in the immediate aftermath of the disaster, along with analyses of WTC toxicants and carcinogens that could be linked to the subsequent cancer development.

### Establishing the tissue bank as a resource for the science community

**Fig. 2:**
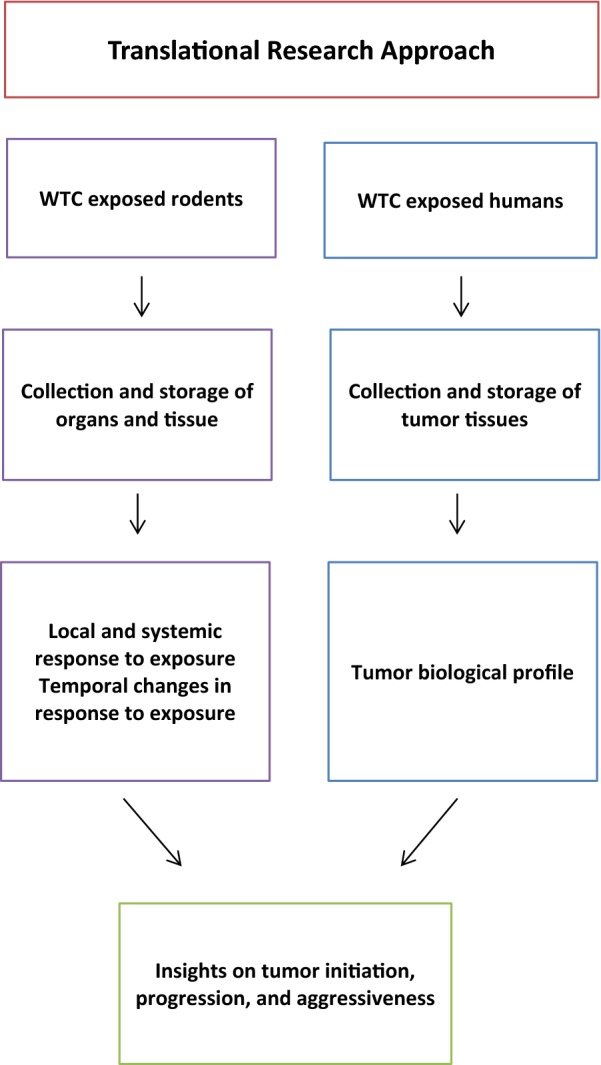
Translational research approach using rodent and human samples

As with the biobank of human cancer tissue [18], the repository of rodent samples is available for use by the scientific community. The animal and human tissue banks are/will be managed in conjunction, and will follow the same process for receiving requests of samples from qualified applicants for research purposes, and for ongoing evaluation of the bank utilization. Comparative research relying on the biological profiles of human and animal exposed tissue samples can be used to study tumor progression and aggressiveness, or systemic inflammatory and immunological responses (Fig. 2). In the future for example, rodent cancer tissue can be used as a proxy to determine if human cancers developing many years after original exposure differ biologically from cancers developed in the immediate aftermath of the WTC disaster, thus helping to disentangle the role of the WTC disaster on cancer occurrence.

### Dissemination

In an effort to reach out to the community, a secure cloud-based website within the Mount Sinai server (http://icahn.mssm.edu/research/epidemiology/capabilities/biorepository-wtc) has been established to act as an information portal modeled on websites previously-developed by our group. The website includes general information about the type of specimens available, procedures and requirements for obtaining tissue, as well as the electronic forms necessary for making tissue requests and inquiries. All scientific publications and presentations involving the tissue bank, and any other activity resulting from use of the tissue bank is recorded in the website.

The human and rodent tissue bank is extensively advertised through partnerships with the WTCHP, CDC, NYU, and the relevant stakeholders and patient advocacy communities [18]. For example, the existence of the tissue bank, as well as any results generated by using the tissue samples is communicated to WTC members during the WTC meetings, is presented to the wider scientific community through posters and scientific articles. In addition, a full page of the Institute for Translational Epidemiology printed brochure has been dedicated to the WTC Biobank. The brochure is currently distributed at major scientific meetings focusing on cancer and epidemiology, and has served as a way to inform the scientific community about the tissue bank.

## Conclusions

A biobank of tissue from rodents exposed to WTC dust has been compiled from NYU and Mount Sinai to complement the human biobank of cancer tissue from WTC first responders. This enhanced tissue bank represents an important and available resource for the scientific community allowing for high impact studies on environmental exposures and cancer etiology, outcomes, and gene-environment interaction in the unique population of WTC responders and WTC-dust induced rodents.

## Data Availability

The datasets generated during the current study are not publicly available but de-identified and anonymized information is potentially available on reasonable request.

## Abbreviations

CV: cardiovascular
CDC: centers for disease control and prevention
DNA: deoxyribonucleic acid
FDNY: Fire Department of the City of New York
IACUC: Institutional Animal Care and Use Committee
ISO: isofluorane
NYU: New York University
PAH: polycyclic aromatic hydrocarbons
PBMC: peripheral blood mononuclear cells
PBS: phosphate-buffered saline
PCB: polychlorinated biphenyls
PTEN: phosphatase and tensin homologue
RNA: ribonucleic acid
SHR: spontaneously hypertensive rats
WTC: World Trade Center
WTCHP: World Trade Center Health Program
